# Transcriptome analyses describe the consequences of persistent HIF-1 over-activation in *Caenorhabditis elegans*

**DOI:** 10.1371/journal.pone.0295093

**Published:** 2024-03-22

**Authors:** Dingxia Feng, Long Qu, Jo Anne Powell-Coffman

**Affiliations:** 1 Department of Genetics, Development and Cell Biology, Iowa State University, Ames, Iowa, United States of America; 2 Department of Statistics, Iowa State University, Ames, Iowa, United States of America; INSERM U869, FRANCE

## Abstract

Metazoan animals rely on oxygen for survival, but during normal development and homeostasis, animals are often challenged by hypoxia (low oxygen). In metazoans, many of the critical hypoxia responses are mediated by the evolutionarily conserved hypoxia-inducible transcription factors (HIFs). The stability and activity of HIF complexes are strictly regulated. In the model organism *C*. *elegans*, HIF-1 stability and activity are negatively regulated by VHL-1, EGL-9, RHY-1 and SWAN-1. Importantly, *C*. *elegans* mutants carrying strong loss-of-function mutations in these genes are viable, and this provides opportunities to interrogate the molecular consequences of persistent HIF-1 over-activation. We find that the genome-wide gene expression patterns are compellingly similar in these mutants, supporting models in which RHY-1, VHL-1 and EGL-9 function in common pathway(s) to regulate HIF-1 activity. These studies illuminate the diversified biological roles played by HIF-1, including metabolism and stress response. Genes regulated by persistent HIF-1 over-activation overlap with genes responsive to pathogens, and they overlap with genes regulated by DAF-16. As crucial stress regulators, HIF-1 and DAF-16 converge on key stress-responsive genes and function synergistically to enable hypoxia survival.

## Introduction

As the electron acceptor during oxidative phosphorylation for energy production, oxygen is vital to all the aerobic organisms. Insufficient oxygen availability not only decreases energy production but also changes the redox environment for cellular biochemical reactions [1, 2]. During normal development and disease states, organisms are often challenged by hypoxia. In metazoans, HIFs have evolutionarily conserved roles in mediating critical transcriptional responses to hypoxia. In mammals, HIF complexes regulate genes functioning in angiogenesis, erythropoiesis and glycolysis to assist hypoxia survival [3–6]. HIF’s function and regulation have potential therapeutic significance in hypoxia-related diseases, such as cancer and stroke. HIF complexes are heterodimers composed of an α subunit and a β subunit, and both subunits are bHLH (basic-helix-loop-helix)-PAS (PER/ARNT/SIM) domain proteins [7]. The human genome encodes three HIFα subunits: HIF-1, -2 and -3α, and three HIFβ/ARNT subunits: HIF-1, -2 and -3β [8]. While HIFβ has multiple bHLH-PAS dimerization partners and is relatively stable and abundant, HIFα is short-lived under well-oxygenated conditions. Thus, HIFα is dedicated to regulate the expression of oxygen-sensitive genes [9–11]. The oxygen-dependent HIFα degradation pathway is conserved and well established. When oxygen levels are high, prolyl hydroxylase domain proteins (PHDs) hydroxylate specific proline residues on HIFα, using oxygen as co-substrate. The hydroxylated HIFα is targeted for proteasomal degradation by an E3 ubiquitin ligase containing the tumor suppressor von Hippel-Lindau (VHL) [2, 10].

HIF and the regulatory network that regulates oxygen-sensitive degradation are conserved and expressed in widely divergent metazoans [12]. HIF and its regulatory system are simplified in the nematode *C*. *elegans*, and this provides opportunities to interrogate this pathway through genetic analyses [13]. In *C*. *elegans*, the single counterparts for HIFα and HIFβ are called HIF-1 and AHA-1, respectively [14–16]. While the *hif-1*α -/- mouse dies by E9.0 with severe vascular defects [17, 18], *C*. *elegans hif-1*(*ia04*) loss-of-function mutants survive and develop normally in room air, although they fail to adapt to hypoxia (0.5% or 1% ambient O_2_) [15, 19, 20]. In *C*. *elegans*, HIF-1 plays diverse biological roles. In addition to regulating hypoxia response, it regulates responses to other stressors, including heat and toxic chemicals (heavy metal cadmium, ethidium bromide, selenium, nanopolystyrene, silver nanoparticles, tunicamycin, tert-butyl hydroperoxide, hydrogen sulfide and hydrogen cyanide), as well as pathogens (*Staphylococcus aureus*, *Vibrio alginolyticus*, enteropathogenic *Escherichia coli*, and *Pseudomonas aeruginosa* PA14 and PAO1) [21–41]. HIF also contributes to the homeostasis of protein and iron [36, 42–47], reproduction, development and apoptosis [39, 48–50], and neural function and animal behaviors [41, 51–60]. Even more interestingly, HIF-1 has been shown to have roles in regulating animal lifespan [43, 44, 61–77].

The PHD-VHL system for HIF stability regulation is conserved and simplified in *C*. *elegans*, too. While humans have three PHDs, *C*. *elegans* has only one counterpart encoded by *egl-9*; and the single VHL homolog in *C*. *elegans* is encoded by *vhl-1* [14].

The two HIF-1 negative regulators: *rhy-1* and *swan-1* have been shown to regulate the expression of some HIF-1 targets [39, 50]. *rhy-1* is a multi-pass transmembrane protein [50], and *swan-1* is a conserved WD repeat scaffold protein [78]. *rhy-1(ok1402)* and *swan-1(ok267)* loss-of-function mutations do not dramatically alter HIF-1 protein levels, but increase expression of some of the genes regulated by HIF-1 [39, 50]. Prior studies [14, 39, 50, 79] support a model in which VHL-1 inhibits HIF-1 stability, EGL-9 inhibits HIF-1 stability and activity, RHY-1 and SWAN-1 suppress HIF-1 activity. Loss-of-function mutations in *egl-9* or *rhy-1* and *swan-1;vhl-1* double mutations cause HIF-1 over-activation. We propose that EGL-9, SWAN-1 and RHY-1 function in common pathway(s) to inhibit HIF-1 activity, and EGL-9 and SWAN-1 may form a complex. This model has been further validated by genetic analyses [80]. Consistent with this model, loss-of-function mutations in *egl-9* or *rhy-1* and *swan-1;vhl-1* double mutations cause an array of similar phenotypes, including egg-laying defects, reduced brood size and resistance to *P*. *aeruginosa* PAO1 [39, 50, 81, 82].

These mutants provide an opportunity to employ multiple genetic backgrounds to over-activate HIF-1 and determine the downstream effects. This also provides some insights to the molecular networks that enable animals to respond to diverse stresses. We answer these questions by comparing the genome-wide transcriptional profiles in these HIF-1 negative regulator mutants.

## Results

### Comparisons of the transcriptional phenotypes of vhl-1(ok161), swan-1(ok267);vhl-1(ok161), egl-9(sa307) and rhy-1(ok1402)

To test our model of HIF-1 regulation and to achieve a richer understanding of the consequences of persistent HIF-1 over-activation, we employed transcriptome analyses to examine the changes in gene expression of animals carrying a deletion in *vhl-1* and animals lacking both *vhl-1* and *swan-1* functions. We also examined animals carrying strong loss-of-function mutation in *egl-9* or *rhy-1*. We reasoned that if, as current models propose, *vhl-1*, *egl-9* and *rhy-*1 acted in common pathway(s) to inhibit HIF-1 activity, then mutations of these genes would cause similar genome-wide gene expression changes.

The complete analysis results for all the probesets on the microarray were provided in S1 Table. Genes up-regulated in *vhl-1(ok161)*, *rhy-1(ok1402)*, *egl-9(sa307)* and *swan-1(ok267);vhl-1(ok161)* double mutants compared to wild-type N2 animals are provided in S2–S5 Tables, respectively. Genes down-regulated in *vhl-1(ok161)*, *rhy-1(ok1402)*, *egl-9(sa307)* and *swan-1(ok267);vhl-1(ok161)* double mutants compared to wild-type N2 animals are provided in S2–S5 Tables, respectively. To further investigate the quality of these datasets, we compared the analysis results to earlier verified gene expressions in these mutants. By RNA blot assays, we and others had demonstrated that *nhr-57*, *cysl-2*/K10H10.2, F22B5.4, *rhy-1*/W07A12.7, *phy-2*, *fmo-2/fmo-*12, *cyp-36A1* and *egl-9* were up-regulated in mutants lacking *vhl-1* or *egl-9* function compared to N2 [41, 83, 84]. And by real-time qRT-PCR, *cysl-2*/K10H10.2 and F22B5.4 had been shown to be up-regulated in *swan-1;vhl-1* and *rhy-1* mutants compared to N2 [39, 50], and *cyp-36A1*, *clec-60*, *clec-52* were up-regulated in *egl-9(sa307)* compared to N2 [38, 41]. Consistent with these results, in our microarray experiment, these genes were up-regulated in the four HIF-1 negative regulator mutants compared to N2 (S2–S5 Tables). The one exception was expected: *rhy-1* mRNA was not over-expressed in the *rhy-1(ok1402)* deletion mutants. Additionally, by real-time qRT-PCR, *lys-5* and *cyp-34A4* had been shown to be down-regulated in *egl-9(sa307)* compared to N2 [38]. In agreement with this, our microarray experiment showed that *lys-5* was down-regulated in *egl-9(sa307)*, *rhy-1(ok1402)* and *swan-1(ok267);vhl-1(ok161)*; and *cyp-34A4* was down-regulated in *egl-9(sa307)* and *swan-1(ok267);vhl-1(ok161)*. In addition, recently, other groups employed RNA-seq to examine the transcriptome profiles of *egl-9(sa307)*, *rhy-1(ok1402)* and *vhl-1(ok161)* [80]. Although different techniques and different statistical cutoffs were used to call differentially expressed genes, the differentially expressed genes identified by RNA-seq (fold change ≠ 1, *q*-value < 0.1) and our microarray experiment (fold change ≥ 1.6, *q*-value ≤ 0.05) overlap significantly: the genes identified as differentially expressed genes in *egl-9(sa307)*, *rhy-1(ok1402)* and *vhl-1(ok161)* mutants in the studies described here overlap with the RNA-seq findings by 38%, 39% and 25%, respectively (S10 Table). Taken together, the consistency between our data and prior or concurrent studies were encouraging.

**Fig 1:**
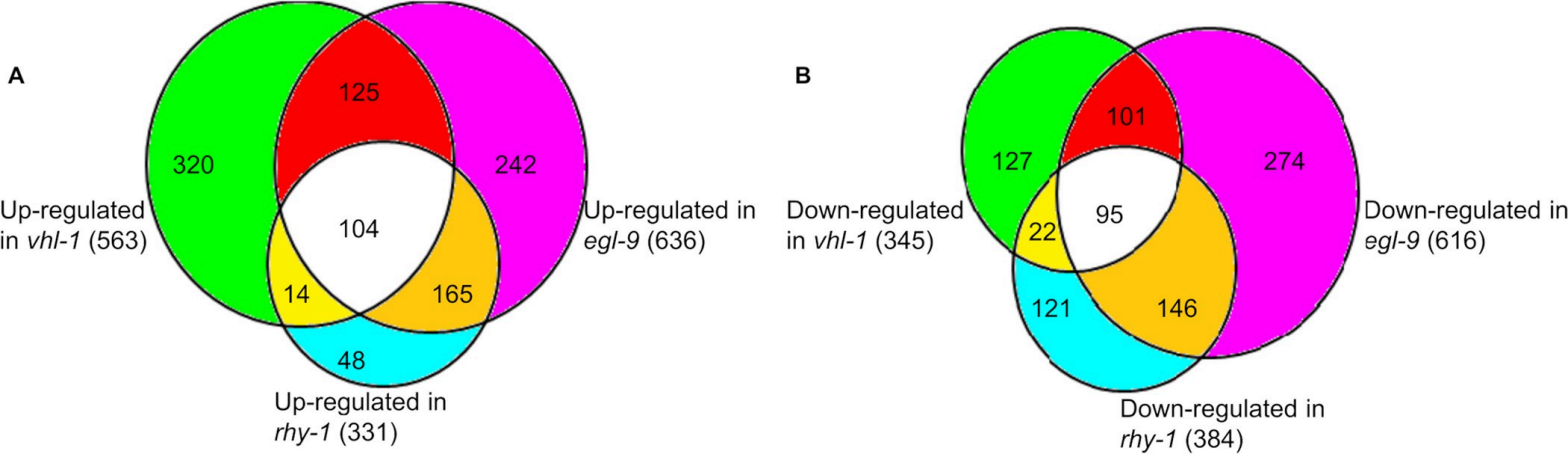
Overlaps of genes differentially expressed in the mutants with persistent HIF-1 high-activity. (A) Numbers of genes up-regulated in *rhy-1*, *egl-9* and *swan-1;vhl-1* loss-of-function mutants, relative to wild-type, and the significant overlaps (*p-*values < 2.2E-16, by Fisher’s exact tests). (B) Numbers of genes down-regulated in *rhy-1*, *egl-9* and *swan-1;vhl-1* loss-of-function mutants, and the significant overlaps (*p-*values < 2.2E-16, by Fisher’s exact tests).

We next focused on the comparisons of gene expression patterns in the three mutants with persistent HIF-1 high-activity: *egl-9(sa307)*, *rhy-1(ok1402)* and *vhl-1(ok161)*. The model that *vhl-1*, *egl-9* and *rhy-1* acted in common pathway(s) to inhibit HIF-1 activity predicted that the gene expression patterns be similar and would reveal genes that were regulated by HIF-1. The overlaps of up-regulated and down-regulated gene sets were analyzed separately. We found 563 genes were up-regulated in *vhl-1(ok161)* (S2 Table), 636 genes were up-regulated in *egl-9(sa307)* (S4 Table), and 331 genes were up-regulated in *rhy-1(ok1402)* (S3 Table). In pair-wise comparisons, 229 genes were commonly up-regulated in *vhl-1(ok161)* and *egl-9(sa307)*. There were also significant overlaps between the genes overexpressed in *rhy-1(ok1402)* and *vhl-1(ok161) (*118 genes) and *rhy-1(ok1402)* and *egl-9(sa307)* (269 genes). In all three comparisons, the overlaps are statistically significant (*p-*values <2.2E-16 by Fisher’s exact tests) (Fig 1A). In sum, the genes that were up-regulated in these three mutants overlapped extraordinarily to illuminate the consequences of long-term HIF-1 over-activation.

We also explored the genes that were expressed at lower levels in the mutants, relative to wild-type animals. We identified 345 genes that were down-regulated in *vhl-1(ok161)* (S6 Table), 616 genes were down-regulated in *egl-9(sa307)* (S8 Table), and 384 genes were down-regulated in *rhy-1(ok1402)* (S7 Table). Pair-wise comparisons revealed significant overlaps in genes downregulated in *vhl-1(ok161)* and *egl-9(sa307)* (196 genes), *rhy-1(ok1402)* and *vhl-1(ok161)*, (117 genes), and *rhy-1(ok1402)* and *egl-9(sa307)* (241 genes). These are illustrated in Fig 1B, and in all three of these comparisons, the overlaps were significant (*p-*values < 2.2E-16, by Fisher’s exact tests). Collectively, these analyses reveal there is a common suite of genes that are expressed at lower levels in these mutants, relative to wild-type, further defining the consequences of HIF-1 over-activation.

In sum, the microarray data revealed that the gene expression patterns in the three HIF-1 high-activity mutants were strikingly similar. This observation supported existing models that RHY-1, VHL-1 and EGL-9 functioned in common pathway(s) to regulate HIF-1 activity.

### Genes regulated by HIF-1 in the HIF-1 negative regulator mutants and under hypoxia

We anticipated some overlap between genes that were mis-regulated in *egl-9*, *vhl-1* or *rhy-1* loss-of-function mutants and genes that had been shown to be induced by hypoxia in a *hif-1-*dependent manner. To test this hypothesis, we asked whether the 104 genes that were up-regulated in all 3 mutants (S11 Table) included genes that had been shown to be positively regulated by HIF-1 in hypoxic conditions [85]. The overlap is striking, as illustrated in S1 Fig, and includes genes for lipid metabolism (ZK550.6 and *gbh-2*), for propionic acid metabolism (*mce-1* and *mmcm-1*), H_2_S and HCN detoxification (*cysl-2*, *ethe-1* and *sqrd-*1), gluconeogenesis (*pck-1*), and protein synthesis regulation (*efk-1*), also for collagen synthesis (*phy-2*) (S13 Table). We also looked for overlaps between the 95 genes that were expressed at lower levels in all three mutants (S13 Table) and genes that had been shown to be repressed by hypoxia in a *hif-1*-dependnet manner [85]. There were fewer genes that were common to all of these gene sets (S1 Fig), and they included T28A11.2 (hypothetical protein), *acdh-2* (Acyl CoA dehydrogenase), and *acs-2* (fatty acid CoA synthetase family) (S14 Table).

### Consequences of persistent HIF-1 over-activation

To more fully understand the consequences of persistent HIF-1 over-activation, we explored the functions the 104 genes that were commonly up-regulated in *vhl-1(ok161)*, *egl-9(sa307)* and *rhy-1(ok1402)* (S11 Table), and the 95 genes that were commonly down-regulated in these three HIF-1 high-activity mutants (S12 Table). The enriched biological terms for genes commonly up-regulated in these three HIF-1 high-activity mutants identified by WormCat at Category 1 level were stress response and metabolism (Table 1). The enriched biological term for these genes at Category 2 level was stress response: pathogen (Table 1). And the enriched biological term for them at Category 3 level was stress response: pathogen: CUB. Genes enriched in these functional categories are listed in Table 1. For genes commonly down-regulated in the three HIF-1 high activity mutants, there was no enriched biological term at Category 1 level with Bonferroni false discovery rate setting at 0.01. The enriched biological term for these genes at Category 2 level were: signaling: hedgehog-like and metabolism: lipid (Table 2). And the enriched biological term for them at Category 3 level were: signaling: hedgehog-like and unassigned: regulated by multiple stresses (Table 2). Genes enriched in each functional category are listed in Table 2.

**Table 1:** Enriched biological terms for genes commonly up-regulated in the three mutants with persistent HIF-1 high activity.

Biological term	Count	Bonferroni FDR	Genes
Cat1: stress response	17	3.40E-07	*gst-19*, *hsp-12*.*3*, *mtl-1*, *nhr-57*, *sip-1*, *skn-1*, C32H11.4, *dod-24*, *cyp-36A1*, *sqrd-1*, *irg-4*, F55G11.8, *sysm-1*, Y43C5A.3, *clec-60*, *clec-52*, *clec-67*
Cat1: Metabolism	23	9.23E-07	*gbh-2*, *tag-38*, *cysl-1*, *ethe-1*, *mce-1*, F26H9.5, *mpst-3*, *dut-1*, *cysl-2*, *sodh-1*, T04A11.1, *aldo-1*, VF13D12L.3, *oac-54*, ZK550.6, *mmcm-1*, *nit-1*, *pcca-1*, *pccb-1*, *cysl-3*, *pck-1*, Y53G8B.2, *tyms-1*
Cat2: stress response: pathogen	7	6.78E-06	*nhr-57*, C32H11.4, *dod-24*, *irg-4*, F55G11.8, *sysm-1*, Y43C5A.3
Cat3: Stress response: pathogen: CUB	3	2.71E-04	C32H11.4, *irg-4*, F55G11.8

**Table 2:** Enriched biological terms for genes commonly down-regulated in the three mutants with persistent HIF-1 high activity.

Biological term	Count	Bonferroni FDR	Genes
Cat2: signaling: hedgehog-like	6	6.36E-07	*grd-11*, *grl-27*, *grl-30*, *ptr-3*, *ptr-23*, *wrt-8*
Cat2: metabolism: lipid	9	6.39E-05	*dhs-25*, *fat-7*, *lbp-5*, *acs-2*, *oac-20*, *acdh-2*, W02F12.2, *gba-4*, *ltah-1*.*2*
Cat3: unassigned: regulated by multiple stresses	22	1.47E-07	*appg-2*, F30F8.5, K01D12.8, *idpp-4*, M03B6.1, C04E12.2, C12D5.9, D1044.1, F15E6.4, F22F4.4, K09C6.9, *cpg-22*, T15B7.1, T20D4.11, T25E4.1, T28A11.2, T28A11.16, Y54G2A.10, Y69A2AR.25, *spp-31*, K08E4.7, C04G6.13
Cat3: signaling: hedgehog-like	6	6.36E-07	*grd-11*, *grl-27*, *grl-30*, *ptr-3*, *ptr-23*, *wrt-8*

Genes commonly up-regulated in these three HIF-1 high-activity mutants included those involved in glycolysis/gluconeogenesis, like *aldo-*1 (fructose-1,6-bisphosphate aldolase) and *pck-1* (phosphoenolpyruvate carboxykinase); this would be relevant to HIF-1’s role for hypoxia adaptation. Lipid metabolism genes like *fat-7*, *acs-2* and *acdh-2* were also shown to be mis-regulated in these three mutants, consistent with HIF-1’s role in regulating lipid metabolism [86, 87]. Interestingly, genes up-regulated in the mutants also include genes involved in propionic acid breakdown (*mce-1*, *mmcm-1*, *pcca-1* and *pccb-1*) [88]. Table 1 also shows that *cysl-1*, *cysl-2*, *ethe-1* and *sqrd-*1 were commonly up-regulated in these three mutants. This was consistent with published findings that HIF-1 was required for *C*. *elegans* to survive in hydrogen sulfide (H_2_S) and hydrogen cyanide (HCN) [29–33]. The enrichment of C-type lectin genes *clec-60*, *clec-52* and *clec-67* and nuclear hormone receptor family member *nhr-57* among genes commonly up-regulated in the three HIF-1 high-activity mutants was consistent with prior studies showing that HIF-1 contributes to pathogen response by regulating C-type lectin genes, lysozyme genes and *nhr-57* [36–38]. Other important stress-responsive genes commonly up-regulated in these three mutants were *mtl-1* (metallothionein) for metal detoxification, *gst-19* for phase II detoxification and heat shock protein *hsp-12*.*3*. This might be related to HIF-1’s functions in heavy metal detoxification and heat stress resistance [22].

### Testing whether *nhr-49* hypoxia pathway functions downstream of *vhl-1*, *egl-9* or *rhy-1*

A recent study has demonstrated that the transcription factor NHR-49 acts in parallel to HIF-1 to regulate hypoxia response, and 83 genes were identified as NHR-49-dependent and HIF-1-independent hypoxia inducible genes [89]. We asked whether these 83 NHR-49 targets were differentially expressed in *vhl-1(ok161)*, r*hy-1*(*ok1402*) and *egl-9*(*sa307)* mutants. We found that none of these overlaps were significant (Table 3). This is consistent with models in which HIF-1 and its upstream regulators act in parallel to NHR-49.

**Table 3:** Overlaps between genes differentially expressed in *vhl-1(ok161)*, *egl-9(sa307)* or *rhy-1(ok1402)* and NHR-49-dependent, HIF-1-independent hypoxia-inducible genes.

NHR-49-dependent 83 genes overlap with	# of overlapped genes	Overlapped genes	*p*-value
563 genes up-regulated in *vhl-1*	4	C01B4.7, *fmo-2*, *gba-2*, *ugt-2*	0.26
636 genes up-regulated in *egl-9*	3	*cyp-34A9*, *fmo-2*, *lgc-1*	0.57
331 genes up-regulated in *rhy-1*	3	*cyp-34A9*, *cyp-37B1*, *lgc-1*	0.20
345 genes down-regulated in *vhl-1*	2	K09D9.1, *acs-2*	0.47
616 genes down-regulated in *egl-9*	4	T16G1.4, *acs-2*, *cyp-13A11*, *nhr-65*	0.32
384 genes down-regulated in *rhy-1*	3	*acs-2*, *ugt-2*, *zip-5*	0.26

### Convergence of HIF-1 pathway and pathogen immune response pathways

It had been shown that r*hy-1*(*ok1402*), *egl-9*(*sa307)* and *swan-1(ok267);vhl-1(ok161)* mutants were resistant to *P*. *aeruginosa* PAO1; and HIF-1 was required for defense against *P*. *aeruginosa* PA14 and PAO1 [36, 37, 39, 41, 81]. It also had been shown that NSY-1/SEK-1/PMK-1 mitogen-activated protein kinase pathway mediated the response to *P*. *aeruginosa* PA14 in *C*. *elegans* [90, 91]. To ask whether these two *P*. *aeruginosa* protective pathways included similar targets, we compared our datasets with published microarray studies that identified the targets of PMK-1 and SEK-1 [90]. We also compared our datasets with genes responding to *P*. *aeruginosa* PA14 infection identified by RNA-seq [92].

We found that genes up-regulated in *vhl-1(ok161)*, *rhy-1(ok1402)*, *egl-9*(*sa307)* and *swan-1(ok267);vhl-1(ok161)* significantly overlapped with genes up-regulated by PMK-1 (Table 4). Genes positively regulated by PMK-1 and HIF-1 included those for immune response, like lysozyme gene *lys-2*, C-type lectin genes (*clec-67* and *clec-85*), ShK-like toxin gene T24B8.5, CUB like domain gene *cld-9*, and hypersensitive to pore-forming toxin gene *hpo-6* (S15 Table). We did not find significant overlaps between genes down-regulated in *vhl-1(ok161)*, *rhy-1(ok1402)*, *egl-9*(*sa307)* or *swan-1(ok267);vhl-1(ok161)* and genes down-regulated by PMK-1 (Table 4).

**Table 4:** Overlaps between genes differentially expressed in *vhl-1(ok161)*, *rhy-1(ok1402)*, *egl-9(sa307)* or *swan-1(ok267);vhl-1(ok161)* and genes responsive to pathogens.

Pair-wise overlaps	# of overlapped genes	*p*-value
563 genes up-regulated in *vhl-1* and 85 genes up-regulated by PMK-1	18	1.01E-10*
331 genes up-regulated in *rhy-1* and 85 genes up-regulated by PMK-1	12	4.66E-8*
636 genes up-regulated in *egl-9* and 85 genes up-regulated by PMK-1	28	6.23E-21*
627 genes up-regulated in *swan-1;vhl-1* and 85 genes up-regulated by PMK-1	38	4.44E-34*
345 genes down-regulated in *vhl-1* and 42 genes down-regulated by PMK-1	1	0.56^NS^
384 genes down-regulated in *rhy-1* and 42 genes down-regulated by PMK-1	3	0.06^NS^
616 genes down-regulated in *egl-9* and 42 genes down-regulated by PMK-1	2	0.42^NS^
623 genes up-regulated in *swan-1;vhl-1* and 42 genes down-regulated by PMK-1	2	0.43^NS^
563 genes up-regulated in *vhl-1* and 101 genes up-regulated by SEK-1	26	2.20E-16*
331 genes up-regulated in *rhy-1* and 101 genes up-regulated by SEK-1	10	1.29E-05*
636 genes up-regulated in *egl-9* and 101 genes up-regulated by SEK-1	27	5.49E-16*
627 genes up-regulated in *swan-1;vhl-1* and 101 genes up-regulated by SEK-1	32	2.30E-22*
345 genes down-regulated in *vhl-1* and 6 genes down-regulated by SEK -1	1	0.11^NS^
384 genes down-regulated in *rhy-1* and 6 genes down-regulated by SEK -1	1	0.12^NS^
616 genes down-regulated in *egl-9* and 6 genes down-regulated by SEK -1	2	0.16 ^NS^
623 genes up-regulated in *swan-1;vhl-1* and 6 genes down-regulated by SEK -1	1	0.19^NS^
563 genes up-regulated in *vhl-1* and 890 genes up-regulated by PA14	110	2.20E-16*
331 genes up-regulated in *rhy-1* and 890 genes up-regulated by PA14	37	3.149E-06*
636 genes up-regulated in *egl-9* and 890 genes up-regulated by PA14	95	2.20E-16*
627 genes up-regulated in *swan-1;vhl-1* and 890 genes up-regulated by PA14	130	2.20E-16*
345 genes down-regulated in *vhl-1* and 803 genes down-regulated by PA14	60	2.20E-16*
384 genes down-regulated in *rhy-1* and 803 genes down-regulated by PA14	50	9.38E-12*
616 genes down-regulated in *egl-9* and 803 genes down-regulated by PA14	78	2.20E-16*
623 genes up-regulated in *swan-1;vhl-1* and 803 genes down-regulated by PA14	83	2.20E-16*
563 genes up-regulated in *vhl-1* and 500 genes up-regulated by Cry5B	42	7.10E-09*
331 genes up-regulated in *rhy-1* and 500 genes up-regulated by Cry5B	8	0.70^NS^
636 genes up-regulated in *egl-9* and 500 genes up-regulated by Cry5B	37	3.10E-5*
627 genes up-regulated in *swan-1;vhl-1* and 500 genes up-regulated by Cry5B	55	1.54E-14*
345 genes down-regulated in *vhl-1* and 584 genes down-regulated by Cry5B	31	3.27E-07*
384 genes down-regulated in *rhy-1* and 584 genes down-regulated by Cry5B	44	3.31E-13*
616 genes down-regulated in *egl-9* and 584 genes down-regulated by Cry5B	93	2.20E-16*
623 genes up-regulated in *swan-1;vhl-1* and 584 genes down-regulated by Cry5B	91	2.20E-16*
563 genes up-regulated in *vhl-1* and 109 genes up-regulated by *Y*. *pestis*	36	2.20E-16*
331 genes up-regulated in *rhy-1* and 109 genes up-regulated by *Y*. *pestis*	10	3.27E-05*
636 genes up-regulated in *egl-9* and 109 genes up-regulated by *Y*. *pestis*	11	0.002^NS^
627 genes up-regulated in *swan-1;vhl-1* and 109 genes up-regulated by *Y*. *pestis*	25	2.1E-15*
345 genes down-regulated in *vhl-1* and 25 genes down-regulated by *Y*. *pestis*	9	4.88E-10*
384 genes down-regulated in *rhy-1* and 25 genes down-regulated by *Y*. *pestis*	10	4.25E-11*
616 genes down-regulated in *egl-9* and 25 genes down-regulated by *Y*. *pestis*	13	2.76E-13*
623 genes up-regulated in *swan-1;vhl-1* and 25 genes down-regulated by *Y*. *pestis*	12	9.10E-12*
563 genes up-regulated in *vhl-1* and 105 genes up-regulated by *S*. *aureus*	11	4.42E-04*
331 genes up-regulated in *rhy-1* and 105 genes up-regulated by *S*. *aureus*	3	0.30^NS^
636 genes up-regulated in *egl-9* and 105 genes up-regulated by *S*. *aureus*	11	0.0012^NS^
627 genes up-regulated in *swan-1;vhl-1* and 105 genes up-regulated by *S*. *aureus*	19	3.35E-09*
345 genes down-regulated in *vhl-1* and 283 genes down-regulated by *S*. *aureus*	10	0.047^NS^
384 genes down-regulated in *rhy-1* and 283 genes down-regulated by *S*. *aureus*	17	1.30E-04*
616 genes down-regulated in *egl-9* and 283 genes down-regulated by *S*. *aureus*	28	7.08E-07*
623 genes up-regulated in *swan-1;vhl-1* and 283 genes down-regulated by *S*. *aureus*	21	8.88E-04*

Genes up-regulated by HIF-1 over-activation also overlapped with genes up-regulated by SEK-1 (Table 4). The overlapped genes included those implicated in immunity and detoxification, such as CUB domain protein genes (*dct-17*, *cld-9* and others), C-type lectin genes (*clec-41*, *clec-66* and *clec-67*), UDP-glucuronosyl transferase *ugt-44*, hypersensitive to pore-forming toxin gene *hpo-6*, and ShK-like toxin gene T24B8.5 (S16 Table). There were no significant overlaps between genes down-regulated in *vhl-1(ok161)*, *rhy-1(ok1402)*, *egl-9*(*sa307)* or *swan-1(ok267);vhl-1(ok161)* and genes down-regulated by SEK-1 (Table 4).

We found genes up-regulated in *vhl-1(ok161)*, *rhy-1(ok1402)*, *egl-9*(*sa307)* and *swan-1(ok267);vhl-1(ok161)* significantly overlapped with genes up-regulated by *P*. *aeruginosa* PA14 infection (Table 4). Genes commonly up-regulated by HIF-1 high activity and *P*. *aeruginosa* PA14 infection included those involved in pathogen response, such as CUB domain protein genes (*dod-22*, F55G11.4, *cld-9* and others), C-type lectin genes (*clec-17*, *clec-4*, *clec-209*, *clec-85*, *clec-41*, *clec-66* and *clec-67*), UDP-glucuronosyl transferase genes (*ugt-13*, *ugt-31*, *ugt-18*, *ugt-16* and *ugt-44*), and ShK-like toxin genes C14C6.5 and *mul-1* (S17 Table). Genes down-regulated in *vhl-1(ok161)*, *rhy-1(ok1402)*, *egl-9*(*sa307)* and *swan-1(ok267);vhl-1(ok161)* also significantly overlapped with genes down-regulated by *P*. *aeruginosa* PA14 infection (Table 4). Genes commonly down-regulated by HIF-1 high activity and *P*. *aeruginosa* PA14 infection included genes for lipid metabolism (like *dhs-25*, *fat-5*, *fat-7*, *gpdh-1*, *acs-1*, *acdh-2*, *gba-4*, *lipl-5* and others) *acox-1*.*2*, *acox-1*.*3*, *pgph-1*, *lipl-1* and *lipl-5*) and amino acid metabolism (like *gcsh-1*, *ddo-2* and *asns-2*). A group of lysozyme genes (like *lys-4*, *lys-6* and *ilys-5*), C-type lectin genes (like *ugt-30*, *ugt-6*, *ugt-64* and *ugt-53*) and UDP-Glucuronosyl transferase genes (like *ugt-30*, *ugt-6*, *ugt-64* and *ugt-53*) were also commonly down-regulated by HIF-1 high-activity and *P*. *aeruginosa* PA14 infection (S17 Table).

We also expected to find overlaps between genes induced by HIF-1 over-activation and those activated by the crystal pore-forming toxin Cry5B, as it had been shown that *egl-9* loss-of-function mutants were resistant to Cry5B in a HIF-1-dependent manner [36]. To address this, we compared our datasets with microarray studies that identified Cry5B-responsive genes [93]. We found that genes up-regulated in *vhl-1(ok161)*, *egl-9*(*sa307)* and *swan-1(ok267);vhl-1(ok161)* significantly overlapped with genes up-regulated by Cry5B, but the overlap between genes up-regulated in *rhy-1(ok1402)* and genes up-regulated by Cry5B was not significant (Table 4). Genes responsive to HIF-1 high-activity and Cry5B included immune responsive genes, such as C-type lectin gene *clec-4*, CUB-like domain genes (*cld-9* and others), cytochrome P450 family gene *cyp-33C8*, alcohol dehydrogenase gene *sodh-1*, hypersensitive to pore-forming toxin gene *hpo-6*, and UDP-glucuronosyl transferase gene *ugt-44* (S18 Table). Genes down-regulated in *vhl-1(ok161)*, *rhy-1(ok1402)*, *egl-9*(*sa307)* and *swan-1(ok267);vhl-1(ok161)* all significantly overlapped with genes down-regulated by Cry5B (Table 4). Genes commonly down-regulated by HIF-1 high-activity and Cry5B included genes for lipid metabolism (like *dhs-25*, *acox-1*.*2*, *acox-1*.*3*, *gpdh-1*, *acs-1*, *pgph-1*, *lipl-1* and *lipl-5*) and amino acid metabolism (like *gcsh-1*, *ddo-2* and *asns-2*). A group of C-type lectin genes were also commonly down-regulated by HIF-1 high-activity and Cry5B, including *clec-10* and *clec-51*.

Recognizing that some of the gene expression changes caused by HIF-1 over-activation were also associated with *Yersinia pestis* response [94], we asked whether genes regulated in the HIF-1 high-activity mutants overlapped with those responsive to *Y*. *pestis*. We found that genes up-regulated in *vhl-1(ok161)*, *rhy-1(ok1402)* and *swan-1(ok267);vhl-1(ok161)* significantly overlapped with genes up-regulated by *Y*. *pestis*, but the overlap between genes up-regulated in *egl-9*(*sa307)* and genes up-regulated by *Y*. *pestis* was not significant (Table 4). Genes commonly up-regulated by *Y*. *pestis* and in *swan-1(ok267);vhl-1(ok161)*, *rhy-1(ok1402)* and *vhl-1 (ok161)* included immune responsive genes, like C-type lectin genes (*clec-60*, *clec-66* and *clec-67*), alcohol dehydrogenase gene *sodh-1*, hypersensitive to pore-forming toxin gene *hpo-6*, and UDP-glucuronosyl transferase gene *ugt-54* (S19 Table). Genes down-regulated in *vhl-1(ok161)*, *rhy-1(ok1402)*, *egl-9*(*sa307)* and *swan-1(ok267);vhl-1(ok161)* all significantly overlapped with genes down-regulated by *Y*. *pestis* infection. Genes commonly down-regulated by HIF-1 high-activity and *Y*. *pestis* infection included *nhr-114* (nuclear hormone receptor), *lys-5* (lysozyme), *fat-5* (fatty acid desaturase) and others (S19 Table).

Finally, we compared our datasets with RNA-seq identified genes regulated by *S*. *aureus* [95]. *S*. *aureus* is another commonly used pathogen in *C*. *elegans* and earlier studies showed that EGL-9 and HIF-1 had roles in *S*. *aureus* resistance [38]. We found that genes up-regulated by *S*. *aureus* infection significantly overlapped with genes up-regulated in *vhl-1(ok161)* and *swan-1(ok267);vhl-1(ok161)*, but the overlaps between genes up-regulated in *rhy-1(ok1402)* or *egl-9*(*sa307)* and genes up-regulated by *S*. *aureus* infection was not significant (Table 4). Genes commonly up-regulated in *vhl-1(ok161)* or *swan-1(ok267);vhl-1(ok161)* and by *S*. *aureus* infection included genes involved in immunity response, like C-type like genes (*clec-187*, *clec-52* and *clec-60*) and UDP-Glucuronosyl transferase genes (*ugt-16*, *ugt-18* and *ugt-44*) (S18 Table). Genes down-regulated by *S*. *aureus* infection significantly overlapped with genes down-regulated in *rhy-1(ok1402)*, *egl-9*(*sa307)* or *swan-1(ok267);vhl-1(ok161)*, but the overlap between genes down-regulated in *vhl-1(ok161)* and genes down-regulated by *S*. *aureus* infection was not significant (Table 4 and S20 Table).

### HIF-1 and DAF-16 function synergistically in hypoxia adaptation

**Fig 2:**
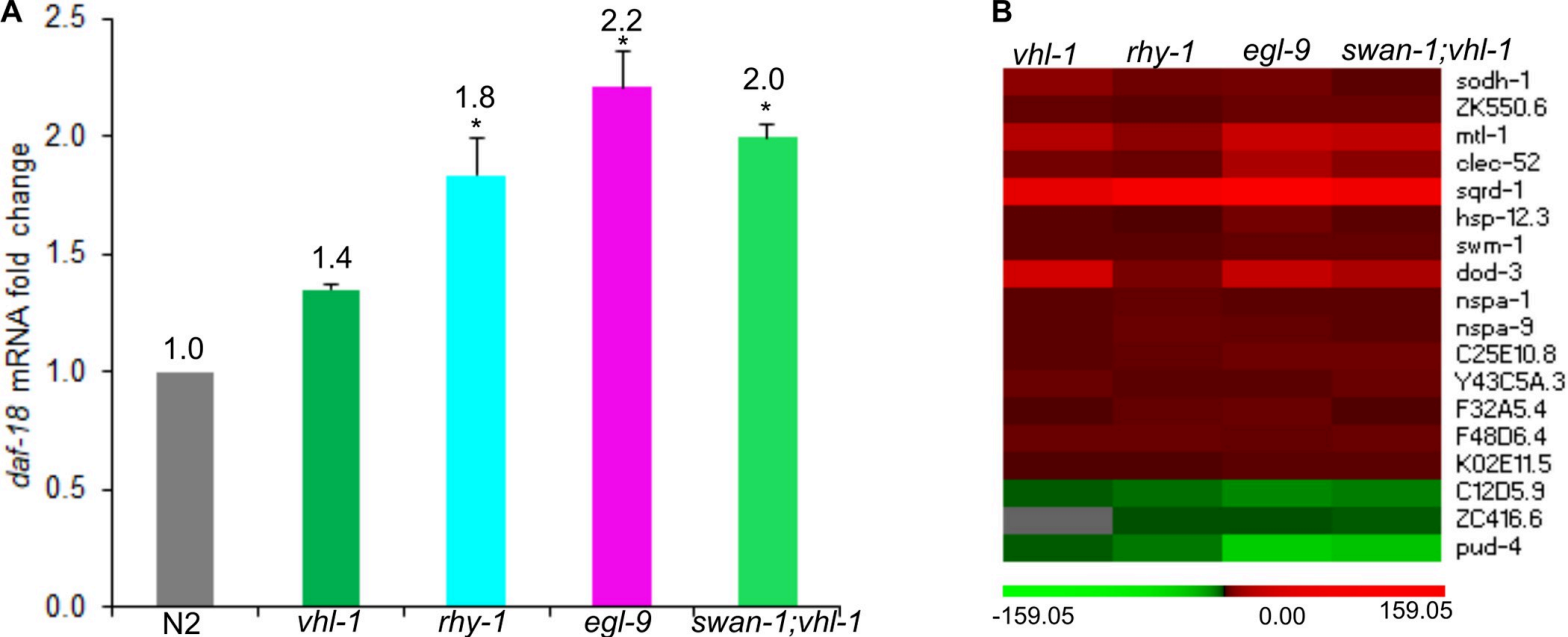
Convergence of the HIF-1 and DAF-16 pathways. (A) *daf-18*, the positive regulator of DAF-16, was up-regulated in *swan-1;vhl-1*, *egl-9* and r*hy-1* mutants (*q-value < 0.05). Error bars are standard errors. (B) A heat map illustrates the expression of 18 genes in *vhl-1*, *swan-1;vhl-1*, *egl-9* and r*hy-1* mutants. These genes were commonly regulated by DAF-16 and in all of these four HIF-1 high-activity mutants. The up-regulated values were represented by red color, down-regulated values were represented by green color. The color intensities correspond to the magnitudes of fold changes relative to N2 as provided in S2–S5 Tables.

We next asked whether the microarray data could illuminate the interaction between HIF-1 and DAF-16. DAF-16 is a forkhead family DNA-binding transcription factor, and it was inhibited by the sole *C*. *elegans* insulin-like receptor DAF-2. In *C*. *elegans*, DAF-16 and HIF-1 both have important roles in metabolism, stress response and longevity [35, 64, 69, 73, 90, 96–102]. However, the mechanism by which these two stress-responsive transcription factors interacted were not well understood. Therefore, it was intriguing to find that *daf-18* mRNA levels were up-regulated in *swan-1(ok267);vhl-1(ok161)*, *egl-9(sa307)* and *rhy-1(ok1402)* mutants compared to wild-type, with fold changes of 2.0, 2.2 and 1.8 respectively (*q*-values were all < 0.05) (S3–S5 Tables and Fig 2A). The mRNA levels of *daf-18* in *vhl-1(ok161)* were not significantly different than in wild-type animals (S2 Table). DAF-18 is homologous to human tumor suppressor PTEN and is an important positive regulator of DAF-16 [103].

We compared genes regulated by DAF-16 [101] with those regulated by HIF-1 over-activation in *vhl-1(ok161)*, *swan-1(ok267);vhl-1(ok161)*, *egl-9(sa307)* or *rhy-1(ok1402)* to see whether they had significant overlaps. There was significant overlap between genes up-regulated in each of these four HIF-1 negative regulator mutants and genes that have been shown to be positively regulated by DAF-16 (Table 5). But there were no significant overlaps between genes down-regulated in these four mutants and genes down-regulated by DAF-16 (Table 5).

**Table 5:** Overlaps between genes differentially expressed in *vhl-1(ok161)*, *rhy-1(ok1402)*, *egl-9(sa307)* or *swan-1(ok267);vhl-1(ok161)* and genes regulated by DAF-16.

Pair-wise overlaps	# of overlapped genes	*p*-value
563 genes up-regulated in *vhl-1* and 251 genes up-regulated by DAF-16	39	< 2.20E-16*
331 genes up-regulated in *rhy-1* and 251 genes up-regulated by DAF-16	26	3.55E-13*
636 genes up-regulated in *egl-9* and 251 genes up-regulated by DAF-16	42	< 2.20E-16*
627 genes up-regulated in *swan-1;vhl-1* and 251 genes up-regulated by DAF-16	32	6.83E-11*
345 genes down-regulated in *vhl-1* and 242 genes down-regulated by DAF-16	5	0.47^NS^
384 genes down-regulated in *rhy-1* and 242 genes down-regulated by DAF-16	11	0.01^NS^
616 genes down-regulated in *egl-9* and 242 genes down-regulated by DAF-16	17	0.003^NS^
623 genes up-regulated in *swan-1;vhl-1* and 242 genes down-regulated by DAF-16	16	0.007^NS^

We further identified 18 genes that were commonly regulated by DAF-16 and in all the four HIF-1 high-activity mutants. Among them, 15 genes were up-regulated by DAF-16 and up-regulated in all the four HIF-1 high-activity mutants; this overlap is significant (*p-*value = 7.26E-12, by Fisher’s exact test). Three genes were down-regulated by DAF-16 and down-regulated in all the four HIF-1 high-activity mutants; this overlap is not significant (*p-*value = 0.11, by Fisher’s exact test). The expression profiles of these 18 genes in *vhl-1(ok161)*, *swan-1(ok267);vhl-1(ok161)*, *egl-9(sa307)* and *rhy-1(ok1402)* are illustrated in a heat map in Fig 2B. This common molecular signature for DAF-16 and HIF-1 activation included increased expressions of stress-responsive and detoxification genes, such as metallothionein gene *mtl-1*, alcohol dehydrogenase gene *sodh-1*, sulfide:quinone reductase gene *sqrd-1*, small heat shock protein gene *hsp-12*.*3*, secreted protease inhibitor genes (*swm-1* and C25E10.8), C-type lectin gene *clec-52*, and others (Fig 2B).

We further investigated the requirement for *daf-16* in moderate hypoxia adaptation (0.5% oxygen, 21°C). Consistent with prior studies, we found that 100% of N2 eggs hatched within 24 hours in hypoxia, and 100% developed to adulthood within 72 hours. By contrast, only 52% of *hif-1(ia04)* mutant eggs hatched within 24 hours in hypoxia, and only 10% developed to adulthood within 72 hours. As shown in Table 6, loss-of-function mutations in *daf-16* also impaired embryogenesis and larval development in hypoxia. For the two *daf-16* loss-of-function alleles (*daf-16(mu86)* and *daf-16 (mgDf50)*) tested, 43–46% of *daf-16*-deficient eggs hatched within 24 hours in hypoxia, and 30–39% developed to adulthood within 72 hours (Table 6). The double mutants of *daf-16* and *hif-1* (*daf-16(mgDf50);hif-1(ia04)*) were most severely affected by hypoxia, only 8% of eggs hatched within 24 hours in hypoxia, and only 0.4% developed to adulthood within 72 hours (Table 6). Note, in room air, the hatched or adult rates for all of these mutant genotypes were the same as those in N2 wild type: 100% of the eggs hatched within 24 hours, and 100% of them developed to adulthood within 72 hours (Table 6). Using the standard provided by Kirienko et al. [104], these data suggest that HIF-1 and DAF-16 acted synergistically to protect *C*. *elegans* from hypoxia insult.

**Table 6:** *hif-1* and *daf-16* functioned synergistically to protect *C*. *elegans* in hypoxia.

Genotype	Treatment	% hatched ± SEM	*p*-value^a^	% survive to adult ± SEM	*p*-value^a^	n^b^
N2	Room air	100.00 ± 0.00	-	100.00 ± 0.00	-	300
	Hypoxia	100.00 ± 0.00	*p* = 0.5	100.00 ± 0.00	*p* = 0.5	300
*hif-1(ia04)*	Room air	100.00 ± 0.00	-	100.00 ± 0.00	-	307
	Hypoxia	52.21 ± 3.31	**p < 0.01	10.25 ± 1.99	**p < 0.01	280
*daf-16(mu86)*	Room air	100.00 ± 0.00	-	100.00 ± 0.00	-	223
	Hypoxia	42.99 ± 2.27	**p < 0.01	30.20 ± 2.11	**p < 0.01	515
*daf-16 (mgDf50)*	Room air	100.00 ± 0.00	-	100.00 ± 0.00	-	222
	Hypoxia	45.52 ± 3.43	**p < 0.01	39.40 ± 3.38	**p < 0.01	240
*daf-16(mgDf50);hif-1(ia04)*	Room air	100.00 ± 0.00	-	100.00 ± 0.00	-	209
	Hypoxia	8.00 ± 1.66	**p < 0.01	0.378 ± 0.376	**p < 0.01	351

^a^ hypoxia against room air for each genotype.

^b^ n is the total number of animals assayed from three biological replicates.

## Discussion

Prior studies have shown that RHY-1, EGL-9 and VHL-1 regulate HIF-1 activity [14, 39, 50, 83], but the extent to which their functions overlap was not fully understood. Here, by employing whole-genome transcriptome analyses, we are able to shed more light on this important regulatory network. The analyses of genes that were differentially expressed in *rhy-1*, *egl-9* and *vhl-1* mutants also extend our understanding of the consequences of multi-generational HIF-1 activation and provides insights to how HIF-1 pathway interacts with other pathways that mediate stress responses.

### Genome-wide gene expression analyses indicate RHY-1, EGL-9 and VHL-1 function in common pathway(s) to regulate HIF-1 activity

Prior assays had shown that selected HIF-1-regulated genes were over-expressed in *egl-9*, *rhy-1* and *vhl-1* mutants. Further, these mutants displayed similar phenotypes in terms of egg-laying defects, reduced brood size and resistance to *P*. *aeruginosa* PAO1 fast killing [39, 50, 81, 82]. These studies indicated that RHY-1, EGL-9 and VHL-1 functioned in common pathway(s) to regulate HIF-1 activity. The transcriptome analyses described here and summarized in Fig 1 fortify this model.

Prior studies also show that *vhl-1*, *egl-9* and *swan-1* have HIF-1-independent functions [38, 41, 78, 83, 105]. In agreement with this, our dataset show that each mutant genotype has its own unique gene expression signature. For example, 38% (242/636) of genes up-regulated in *egl-9(sa307)* are unique to itself and not differentially expressed in *rhy-1(ok1402)* or *vhl-1(ok161)*. Similarly, 57% (320/563) of genes up-regulated in *vhl-1(ok161)* are not differentially expressed in *rhy-1(ok1402)* or *egl-9(sa307)*. Also, 15% (48/331) 11% (35/325) of genes up-regulated in *rhy-1(ok1402)* are not differentially expressed in *egl-9(sa307)* or *vhl-1(ok161)* (Fig 1A). The same conclusion applies to genes down-regulated in these mutants: 44% (= 274/616) of genes down-regulated in *egl-9(sa307)* are not differentially expressed in *rhy-1(ok1402)* or *vhl-1(ok161)*; 37% (= 127/345) 35% (= 212/613) of genes down-regulated in *vhl-1(ok161)* are not differentially expressed in *rhy-1(ok1402)* or *egl-9(sa307)*; and 32% (= 121/384) 31% (= 117/380) of genes down-regulated in *rhy-1(ok1402)* are not differentially expressed in *egl-9(sa307)* or *vhl-1(ok161)* (Fig 1B). Prior studies had provided evidence that NHR-49 acted independently of HIF-1 to regulate hypoxia response [89], and the data provided here support this conclusion as well. As summarized in Table 3, the overlaps between genes regulated by NHR-49 [89] and genes misregulated in *egl-9*, *vhl-1*, or *rhy-1* loss-of-function mutants are not statistically significant. The interplay between HIF-1 and NHR-49 is an intriguing area for future studies.

### The pleiotropic consequences of persistent HIF-1 over-activation account for its diversified biological roles

Misregulation of HIF-1 disrupts multiple facets of *C*. *elegans*’ life, including hypoxia adaptation, hydrogen sulfide and hydrogen cyanide resistance, heat resistance, heavy metal toxicity tolerance, pathogen response, egg-laying defect, brood size and aging [22, 29, 30, 34–36, 38, 39, 44, 50, 61–64, 81, 82]. Our analyses provide a more complete understanding of gene expression changes that underpin these diverse phenotypes. The common set of genes misregulated in *vhl-1(ok161)*, *egl-9(sa307)* and *rhy-1(ok1402)* are involved in the metabolism of sugars, lipids, amino acids, and sulfur. Consistent with this, it have been shown that the metabolomic profiles of various amino acids, carbohydrates, lipids and nucleotides are changed in *egl-9(sa307)* mutants [106]. They also have roles in hypoxia and innate immune response. These various biological processes regulated by persistent HIF-1 over-activation suggest models for why HIF-1 can play a variety of biological roles besides regulating hypoxia response. For example, the up-regulated sulfur metabolism by HIF-1 over-activation is relevant to HIF-1’s roles in hydrogen sulfide and hydrogen cyanide resistance [29, 30, 32–34]. Also, the up-regulated innate immune response by HIF-1 over-activation explains its roles in pathogen resistance [35, 36, 38–41, 81, 104], which is further supported by the overlaps of genes regulated by HIF-1 over-activation with genes responsive to toxic Cry5B, *P*. *aeruginosa* PA14, *S*. *aureus* and *Y*. *pestis* [36, 92, 94, 95], and with genes regulated by PMK-1 and SEK-1, the major players for pathogenic *P*. *aeruginosa* PA14 resistance [91]. We recognized that the methods we used to identify the overlaps between differentially expressed gene lists has its own limitation. When doing genome-wide analyses, researchers rely on statistical cut offs to focus inquiry on the genes for which the statistical data is the strongest. Additional repetitions would no doubt identify more genes as differentially expressed. This dataset provides a starting point for further study.

### HIF-1 interacts with DAF-16 to promote hypoxia adaptation

DAF-16 and HIF-1 are both important stress regulators in *C*. *elegans*. In this study, we demonstrate that DAF-16 and HIF-1 have complicated complementary and overlapping roles in stress response. On one hand, they converge on key stress-responsive genes. On the other hand, they function synergistically to promote hypoxia adaptation. This suggests that DAF-16 is largely required outside the HIF-1 pathway to promote hypoxia resistance. Intriguingly, the mRNA levels of *daf-18*, the positive regulator of DAF-16, are up-regulated in *swan-1(ok267);vhl-1(ok161)*, *egl-9(sa307)* and *rhy-1(ok1402)* mutants. This suggests a mechanism for the crosstalk between HIF-1 and DAF-16: HIF-1 may enhance DAF-16’s function by increasing the expression of *daf-18*. While beyond the scope of the experiments reported here, future studies might explore the ways in which these two important stress-response pathways regulate each other and converge to enable the organism to adapt and survive adverse conditions.

## Materials and methods

### Strains

The wild-type *C*. *elegans* used in this study was N2 Bristol. The loss-of-function mutation alleles used in this study were: LGI: *daf-16(mu86)lf*, *daf-16(MgDf50)lf*; LGII: *rhy-1(ok1402)lf*; LGV: *hif-1(ia04)lf*, *egl-9(sa307)lf*, *swan-1(ok267)lf*; LGX: *vhl-1(ok161)lf*. All the worms were maintained at 21°C using the standard methods [107].

### Gene expression microarray experiment

Randomized complete block design was followed for the microarray experiment, with three biological replicates treated as three blocks. Each block included eight treatments: N2 wild type, N2 wild type with hypoxia treatment, *hif-1*(*ia04)* loss-of-function mutants, *hif-1*(*ia04)* loss-of-function mutants with hypoxia treatment, *vhl-1(ok161)* loss-of-function mutants, *rhy-1(ok1402)* loss-of-function mutants, *egl-9(sa307)* loss-of-function mutants and *swan-1(ok267);vhl-1(ok161)* loss-of-function double mutants. For each treatment, about 1,000 synchronized L4-stage larvae were pooled as one experimental unit to get sufficient RNA for hybridization. Total RNA isolation was performed using Trizol (Invitrogen) and RNeasy Mini Kit (Qiagen). RNA quality was checked with an Agilent 2100 BioAnalyzer (Agilent Technologies). The RNA integrity numbers (RINs) for all the samples used in this study were greater than 9.0. The total RNA isolated from one experimental unit was hybridized onto one Affymetrix GeneChip® C. elegans Genome array (Affymetrix, part number 900383). Probe synthesis, labeling, hybridization, washing, staining and scanning were performed by the GeneChip facility at Iowa State University. In brief, the total RNA was synthesized to biotin-labeled aRNA using the GeneChip® 3’ IVT Express Kit (Affymetrix, part number 901229) and hybridized to the array. The arrays were washed and stained in the GeneChip® fludics station 450 and scanned with GeneChip® scanner 3000 7G. The Affymetrix® GeneChip® Command Console™ (AGCC) software was used to generate probe cell intensity data (.CEL) files. The resulting CEL files were normalized and summarized using the robust multichip average (RMA) algorithm [108] in R package (R Core Team, Vienna, Austria, 2016). An analysis of variance (ANOVA) model was then fitted to the summarized expression measures, with the block (three levels) and the treatment (eight levels) treated as fixed effect factors following the experimental design. Residual model diagnostics identified no severe violations of the model assumptions. Linear contrasts of treatment means were tested using the general F-test. To account for multiplicities of hypothesis testing, conservative estimates of false discovery rates (FDRs) were calculated according to the *q*-value procedure of Storey and Tibshirani [109]. Differentially expressed probesets were defined as *q*-value ≤ 0.05 and fold change ≥ 1.6. Probesets were converted to genes using the Affymetrix annotation file “Celegans.na36.annot.csv”. To deal with redundancy and count the number of unique genes detected on the array, we kept one probeset per gene and one gene per probeset. In this way, the total number of unique genes detected on the array was 18, 011. For the purpose of reference, the original complete lists of gene(s) annotated to each probeset were kept in S1–S8 Tables. The complete analysis results for all the conditions (N2 wild type, N2 wild type with hypoxia treatment, *hif-1*(*ia04)* loss-of-function mutants, *hif-1*(*ia04)* loss-of-function mutants with hypoxia treatment, *vhl-1(ok161)* loss-of-function mutants, *rhy-1(ok1402)* loss-of-function mutants, *egl-9(sa307)* loss-of-function mutants and *swan-1(ok267);vhl-1(ok161)* loss-of-function double mutants) and all the probesets on the microarray are provided in S1 Table. Gene expression changes in N2 wild type animals and *hif-1(ia04)* mutants under hypoxia and room air, and the comparison of gene expressions under hypoxia and in the HIF-1 negative regulator mutants, also the expression of HIF-1 direct targets identified by CHIP-seq have been presented in a related study [85]. This study focuses on the genes expression changes in *vhl-1(ok161)*, *egl-9(sa307)*, *rhy-1(ok1402)* and *swan-1(ok267);vhl-1(ok161)* double mutants. The microarray raw and probeset summary data had been deposited to NCBI’s Gene Expression Omnibus, the accession number was GSE228851.

### Gene function annotation and enrichment analyses

WormCat online tool (www.wormcat.com) [110] was used to annotate the enriched biological terms associated with microarray-selected genes. The enriched biological terms were at Bonferroni false discovery rate cut off of 0.01.

### Heat maps

Heat maps for gene expression profiles were generated using the PermutMatrix graphical analysis program [111, 112]. Average linkage clustering was performed with the fold changes compared to N2. Green color represented negative values, and red color represented positive values. The color intensities corresponded to the magnitudes of fold changes. Other parameters were set as default.

### Gene lists overlap testing

Fisher’s exact test was performed to test whether the overlap between two gene lists was significant or not. The total number of 18, 011 genes detected on the microarray was used as the population size. The significant overlap is at *p*-value < 0.001.

### Hypoxia development and survival assays

For each genotype, the room air and hypoxia treatments were performed in parallel at 21°C. For each treatment, 20 young adults (one day after L4 molt) were used as parents to lay eggs on one NGM plate seeded with OP50 for 30 minutes. After counting the eggs laid, the plates were kept in room air or put into a sealed plexiglass chamber with constant hypoxic gas flow. Compressed air and 100% nitrogen were mixed to achieve 0.5% oxygen, and gas flow was controlled by an oxygen sensor [84]. After 24 hours, the un-hatched eggs were counted for both treatments. After that, the plates for both treatments were maintained in room air. The adult worms were counted 72 hours after the eggs had been laid. The data collection time points were set to match the development rate of N2 eggs in room air: they hatch within 24 hours and reach adulthood within 72 hours. The experiments were performed with three biological replicates. To test the effect of hypoxia on animal development and survival, the binary hatched *vs*. un-hatched or adult *vs*. non-adult data were analyzed for each genotype by fitting a generalized linear model using a logit link function with JMP 9 statistical software (SAS Institute Inc., Cary, NC, 2010). The replicate (three levels) and the treatment (two levels) were used as factors in the model.

## Supporting information

S1 Table Gene expression for all the probesets under hypoxia and in the HIF-1 negative regulator mutants. (XLSX)

S2 Table Genes up-regulated in *vhl-1*. (XLSX)

S3 Table Genes up-regulated in *rhy-1*. (XLSX)

S4 Table Genes up-regulated in *egl-9*. (XLSX)

S5 Table Genes up-regulated in *swan-1;vhl-1*. (XLSX)

S6 Table Genes down-regulated in *vhl-1*. (XLSX)

S7 Table Genes down-regulated in *rhy-1*. (XLSX)

S8 Table Genes down-regulated in *egl-9*. (XLSX)

S9 Table Genes down-regulated in *swan-1;vhl-1*. (XLSX)

S10 Table Overlaps between differentially expressed genes (DEGs) identified by RNA-seq and microarray. (XLSX)

S11 Table Genes commonly up-regulated in *vhl-1*, *rhy-1* and *egl-9*. (XLSX)

S12 Table Genes commonly down-regulated in *vhl-1*, *rhy-1* and *egl-9*. (XLSX)

S13 Table Genes positively regulated by HIF-1 under hypoxia and in the three HIF-1 negative regulators. (XLS)

S14 Table Genes negatively regulated by HIF-1 under hypoxia and in the three HIF-1 negative regulators. (XLS)

S15 Table Genes upregulated by PMK-1 and HIF-1. (XLSX)

S16 Table Genes upregulated by SEK-1 and HIF-1. (XLSX)

S17 Table Genes regulated by HIF-1 and *P*. *aeruginosa* PA14. (XLSX)

S18 Table Genes regulated by Cry5B and HIF-1. (XLSX)

S19 Table Genes regulated by *Yersinia pestis* and HIF-1. (XLSX)

S20 Table Genes regulated by *S*. *aureus* and HIF-1. (XLSX)

S1 Fig Overlaps between genes regulated in the HIF-1 negative regulator mutants and genes regulated by HIF-1 under hypoxia. (PPTX)
