# Supplementary material for: Transcriptome analyses describe the consequences of persistent HIF-1 over-activation in Caenorhabditis elegans
Source: PLoS One. 2024 Mar 22;19(3):e0295093. doi: 10.1371/journal.pone.0295093 (PMC10959373; doi:10.1371/journal.pone.0295093)
Supplement: S1 Fig — (PPTX) [file pone.0295093.s021.pptx]

## Slide 1
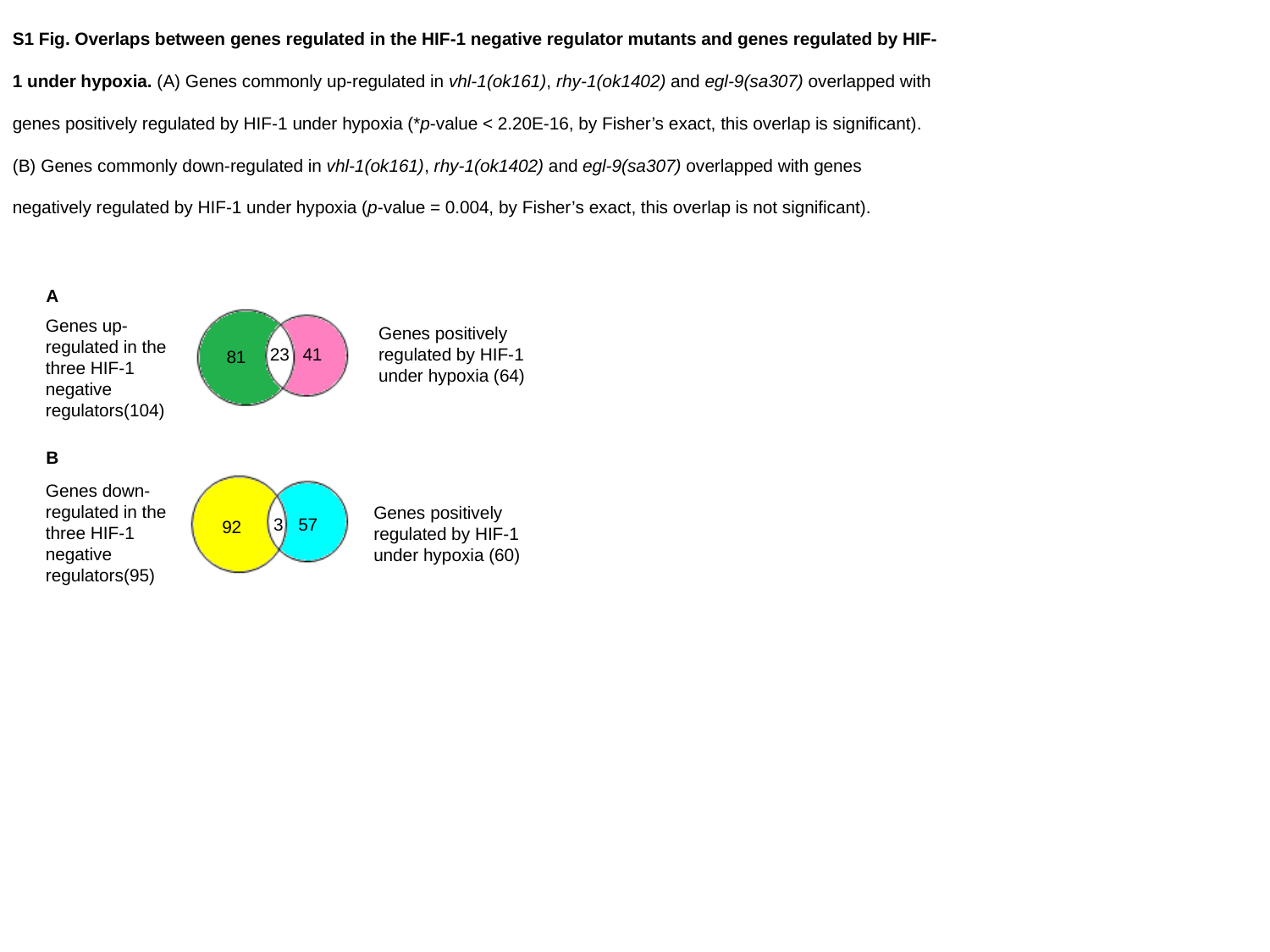

S1 Fig. Overlaps between genes regulated in the HIF-1 negative regulator mutants and genes regulated by HIF-1 under hypoxia. (A) Genes commonly up-regulated in vhl-1(ok161), rhy-1(ok1402) and egl-9(sa307) overlapped with genes positively regulated by HIF-1 under hypoxia (*p-value < 2.20E-16, by Fisher’s exact, this overlap is significant). (B) Genes commonly down-regulated in vhl-1(ok161), rhy-1(ok1402) and egl-9(sa307) overlapped with genes negatively regulated by HIF-1 under hypoxia (p-value = 0.004, by Fisher’s exact, this overlap is not significant).
A
Genes up-regulated in the three HIF-1 negative regulators(104)
Genes positively regulated by HIF-1 under hypoxia (64)
41
23
81
B
Genes down-regulated in the three HIF-1 negative regulators(95)
Genes positively regulated by HIF-1 under hypoxia (60)
57
3
92
